# Poly[{μ_10_-[(phosphono­meth­yl)imino­dimethyl­ene]diphospho­nato}dithallium(I)]

**DOI:** 10.1107/S1600536809031006

**Published:** 2010-07-03

**Authors:** Khodayar Gholivand, Ali Reza Farrokhi

**Affiliations:** a Department of Chemistry, Tarbiat Modares University, PO Box 14115-175 Tehran, Iran

## Abstract

The title compound, [Tl_2_(C_3_H_10_NO_9_P_3_)]_*n*_, a Tl^I^ organic–inorganic hybrid complex, was synthesized by the reaction of nitrilo­tris(methyl­enephospho­nic acid) with thallium(I) nitrate. There are two types of Tl^+^ ions in the complex, with coordination numbers of eight and seven and with stereochemically active and inactive lone-pair electrons, respectively. In the crystal, the doubly deprotonated ligands form two-dimensional hydrogen-bonded layers through O—H⋯O hydrogen bonds. The NH group is involved in a trifurcated intra­molecular hydrogen bond. Coordination of the phospho­nate ligands to the Tl^+^ ions creates a three-dimensional structure.

## Related literature

For related metal phospho­nate complexes of the same ligand, see: Sharma *et al.* (2001 ▶).
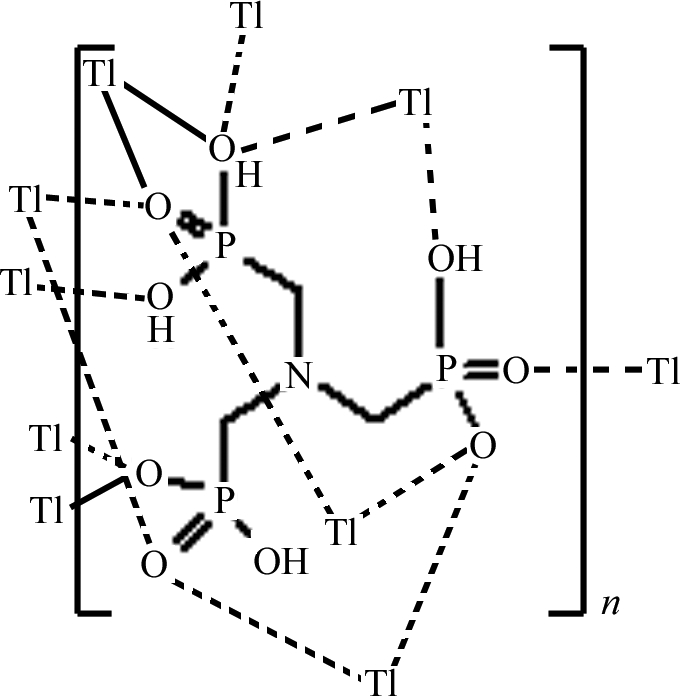


## Experimental

### 

#### Crystal data

[Tl_2_(C_3_H_10_NO_9_P_3_)]
*M* *_r_* = 705.77
Triclinic, 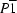

*a* = 7.9236 (6) Å
*b* = 8.0932 (6) Å
*c* = 10.9136 (8) Å
α = 81.422 (1)°
β = 79.023 (1)°
γ = 68.085 (1)°
*V* = 635.06 (8) Å^3^
*Z* = 2
Mo *K*α radiation
μ = 25.76 mm^−1^
*T* = 100 K
0.16 × 0.14 × 0.10 mm

#### Data collection

Bruker APEXII CCD area-detector diffractometer
Absorption correction: numerical (*XPREP*; Bruker, 2007 ▶) *T*_min_ = 0.104, *T*_max_ = 0.183
6627 measured reflections
2744 independent reflections
2437 reflections with *I* > 2σ(*I*)
*R*_int_ = 0.029

#### Refinement

*R*[*F* ^2^ > 2σ(*F* ^2^)] = 0.025
*wR*(*F* ^2^) = 0.055
*S* = 1.05
2744 reflections
163 parameters
12 restraints
H-atom parameters constrained
Δρ_max_ = 1.72 e Å^−3^
Δρ_min_ = −1.83 e Å^−3^

### 

Data collection: *APEX2* (Bruker, 2007 ▶); cell refinement: *SAINT* (Bruker, 2007 ▶); data reduction: *SAINT*; program(s) used to solve structure: *SHELXS97* (Sheldrick, 2008 ▶); program(s) used to refine structure: *SHELXL97* (Sheldrick, 2008 ▶); molecular graphics: *SHELXTL* (Sheldrick, 2008 ▶); software used to prepare material for publication: *SHELXTL*.

## Supplementary Material

Crystal structure: contains datablocks I, global. DOI: 10.1107/S1600536809031006/su2128sup1.cif

Structure factors: contains datablocks I. DOI: 10.1107/S1600536809031006/su2128Isup2.hkl

Additional supplementary materials:  crystallographic information; 3D view; checkCIF report

## Figures and Tables

**Table 1:** Hydrogen-bond geometry (Å, °)

*D*—H⋯*A*	*D*—H	H⋯*A*	*D*⋯*A*	*D*—H⋯*A*
N1—H1*N*⋯O3	0.87	2.39	2.882 (5)	116
N1—H1*N*⋯O5	0.87	2.53	2.957 (6)	111
N1—H1*N*⋯O8	0.87	2.19	2.837 (7)	131
O1—H1*O*⋯O1^i^	0.82	1.78	2.504 (8)	147
O2—H2*O*⋯O2^ii^	0.82	1.68	2.497 (7)	171
O6—H6*O*⋯O9^iii^	0.82	1.67	2.484 (6)	171
O7—H7*O*⋯O4^iv^	0.82	1.70	2.521 (6)	180

Symmetry codes: (i) 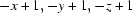; (ii) 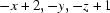; (iii) 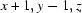; (iv) 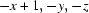.

**Fig. 1.**
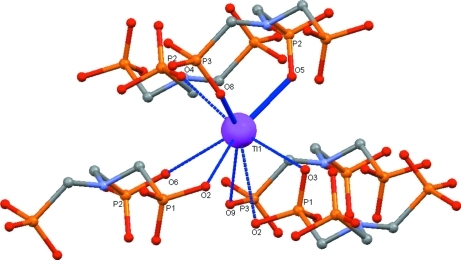
Environment of the Tl1 atom.

**Fig. 2.**
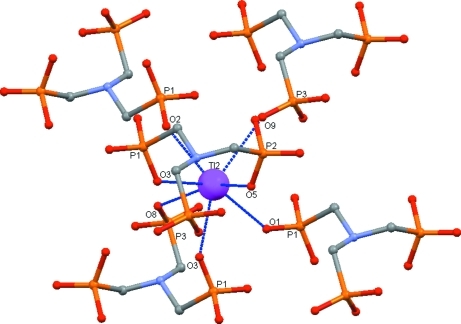
Environment of the Tl2 atom.

**Fig. 3.**
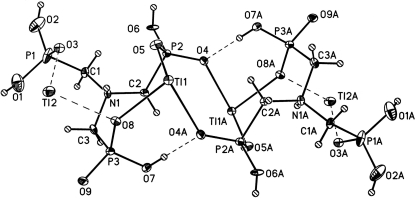
The conformation of the doubly deprotonated nitrilotris(methylenephosphonate) ligand.

**Fig. 4.**
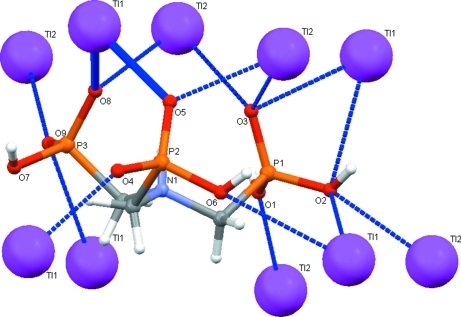
Each H4L2- dianion links to five Tl1 and five Tl2 ions.

**Fig. 5.**
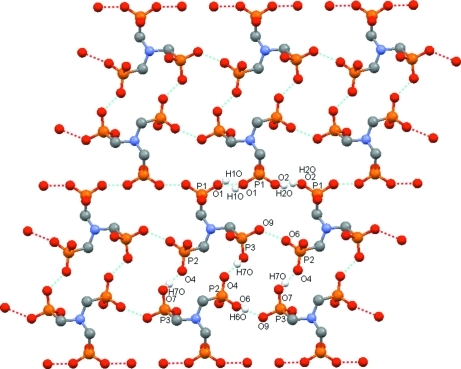
Two-dimensional hydrogen-bonded layers formed via O—H···O hydrogen bonds involving hydroxyl groups.

**Fig. 6.**
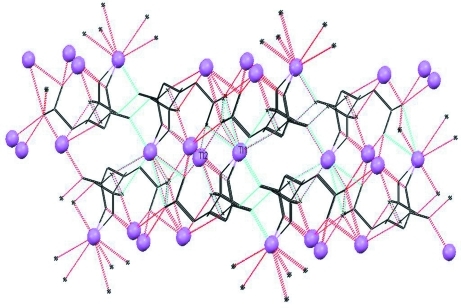
The three-dimensional structure resulting from the coordination of the ligand to the metal ions in the interlayer space.

**Fig. 7.**
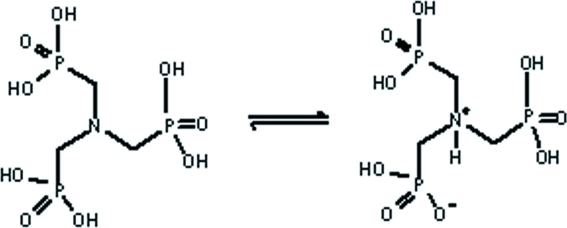
Reaction scheme.

## supplementary crystallographic information

### Comment

Metal phosphonate complexes of the ligand nitrilotris(methylenephosphonic acid (H6L), giving two different types of 1:1 (M/L) metal phosphonate complexes, M[NH(CH_2_PO_3_H)_3_(H_2_O)_3_](M = Mn, Co, Ni, Cu, Zn, Cd), with two-dimensional hydrogen-bonded layered structures, have been prepared previously by (Sharma *et al.*, 2001).

In the structure of the title compound, synthesized by the reaction of the same ligand with thallium(I) nitrate, the nitrilotris(methylenephosphonate) (H4L^2-^) group is doublely deprotonated and two different environments for the Thallium atoms are observed, as shown in Figs. 1 and 2. Eight O-atoms of five H4L^2-^ phosphonate ligands are coordinated to the Tl1 ion (Fig. 1), while seven oxygen atoms of five H4L^2-^ phosphonate ligands are coordinated to the Tl2 ion (Fig. 2).

The conformation of the doublely deprotonated nitrilotris(methylenephosphonate) ligand is illustrated in Fig. 3. The hydrogen atoms on atoms O1 and O2 are positionally disordered with relative occupancies of 0.5:0.5. Each H4L^2-^ dianion links to five Tl1 and five Tl2 ions (Fig. 4).

The doublely deprotonated H4*L*^2-^ ligand forms two-dimensional hydrogen bonded layers *via* O—H···O hydrogen bonds involving hydroxyl groups O1, O2, O6 and O7 (Table 1 and Fig. 5). Atoms O3, O5 and O8 do not contribute in this type of hydrogen bond, rather they coordinate only to the Tl^+^ions. The NH group is involved in a 4-centre trifurcated intramolecular hydrogen bond with O-atoms O3, O5 and O8 (Table 1). Coordination of the ligand to the metal ions in the interlayer space creates a three-dimensional structure (Fig. 6).

### Experimental

Thallium(I)nitrate (0.133 g, 0.5 mmol) was added in several portions to a solution of nitrilotris(methylenephosphonicacid) (H6L) (0.104 g, 0.35 mmol) in 12 ml of a deionized water-ethanol mixture (3:5). The solution was stirred for seven days and a white precipitate was obtained. This was filtered off and recrystallized from deionized water at rt. Colorless prism-like crystals of the title compound were obtained in 68% yield (based on the Tl atom). Elemental analysis for Tl_2_(H_4_L), C_3_H_10_NO_9_P_3_Tl_2_:*C*, 5.06; H, 1.36; N, 2.03%. Calc.: C, 5.10; H, 1.41; N, 1.98%.

### Refinement

The NH and OH H-atoms were located in a difference electron-density map and were refined with distance restraints: O—H = 0.82 (2) and N—H = 0.86 (2) Å, with *U*_iso_(H) = 1.2*U*_eq_(N,O). The C-bound H atoms were positioned geometrically and refined using a riding model: C—H = 0.99 Å, with *U*_iso_(H) = 1.2 times *U*_eq_(C).

### Crystal data

**Table:**

[Tl_2_(C_3_H_10_NO_9_P_3_)]	*Z* = 2
*M**_r_* = 705.77	*F*(000) = 628
Triclinic, *P*1	*D*_x_ = 3.691 Mg m^−^^3^
Hall symbol: -P 1	Mo *K*α radiation, λ = 0.71073 Å
*a* = 7.9236 (6) Å	Cell parameters from 266 reflections
*b* = 8.0932 (6) Å	θ = 3–26°
*c* = 10.9136 (8) Å	µ = 25.76 mm^−^^1^
α = 81.422 (1)°	*T* = 100 K
β = 79.023 (1)°	Prism, colorless
γ = 68.085 (1)°	0.16 × 0.14 × 0.10 mm
*V* = 635.06 (8) Å^3^	

### Data collection

**Table:**

Bruker APEXII CCD area-detector diffractometer	2744 independent reflections
Radiation source: fine-focus sealed tube	2437 reflections with *I* > 2σ(*I*)
graphite	*R*_int_ = 0.029
ω scans	θ_max_ = 27.0°, θ_min_ = 1.9°
Absorption correction: numerical (*XPREP*; Bruker, 2007)	*h* = −10→10
*T*_min_ = 0.104, *T*_max_ = 0.183	*k* = −10→10
6627 measured reflections	*l* = −13→13

### Refinement

**Table:**

Refinement on *F*^2^	Primary atom site location: structure-invariant direct methods
Least-squares matrix: full	Secondary atom site location: difference Fourier map
*R*[*F*^2^ > 2σ(*F*^2^)] = 0.025	Hydrogen site location: inferred from neighbouring sites
*wR*(*F*^2^) = 0.055	H-atom parameters constrained
*S* = 1.05	*w* = 1/[σ^2^(*F*_o_^2^) + (0.0225*P*)^2^ + 1.104*P*] where *P* = (*F*_o_^2^ + 2*F*_c_^2^)/3
2744 reflections	(Δ/σ)_max_ = 0.003
163 parameters	Δρ_max_ = 1.72 e Å^−^^3^
12 restraints	Δρ_min_ = −1.83 e Å^−^^3^

### Special details

**Table:**

Geometry. All e.s.d.'s (except the e.s.d. in the dihedral angle between two l.s. planes) are estimated using the full covariance matrix. The cell e.s.d.'s are taken into account individually in the estimation of e.s.d.'s in distances, angles and torsion angles; correlations between e.s.d.'s in cell parameters are only used when they are defined by crystal symmetry. An approximate (isotropic) treatment of cell e.s.d.'s is used for estimating e.s.d.'s involving l.s. planes.
Refinement. Refinement of *F*^2^ against ALL reflections. The weighted *R*-factor *wR* and goodness of fit *S* are based on *F*^2^, conventional *R*-factors *R* are based on *F*, with *F* set to zero for negative *F*^2^. The threshold expression of *F*^2^ > σ(*F*^2^) is used only for calculating *R*-factors(gt) *etc*. and is not relevant to the choice of reflections for refinement. *R*-factors based on *F*^2^ are statistically about twice as large as those based on *F*, and *R*- factors based on ALL data will be even larger.

### Fractional atomic coordinates and isotropic or equivalent isotropic displacement parameters (Å^2^)

**Table:**

	*x*	*y*	*z*	*U*_iso_*/*U*_eq_	Occ. (<1)
Tl1	0.29076 (3)	−0.05875 (3)	0.25484 (2)	0.01064 (7)	
Tl2	0.23248 (3)	0.24843 (3)	0.54672 (2)	0.01703 (8)	
P1	0.7521 (3)	0.2462 (3)	0.42352 (16)	0.0201 (4)	
P2	0.7554 (2)	−0.0669 (2)	0.10302 (14)	0.0090 (3)	
P3	0.2557 (2)	0.4329 (2)	0.20298 (14)	0.0091 (3)	
O1	0.6684 (8)	0.4387 (7)	0.4597 (5)	0.0331 (13)	
H1O	0.5753	0.4894	0.5072	0.040*	0.50
O2	0.9424 (7)	0.1622 (8)	0.4631 (5)	0.0311 (12)	
H2O	0.9828	0.0586	0.4936	0.037*	0.50
O3	0.6291 (6)	0.1391 (5)	0.4655 (4)	0.0119 (9)	
O4	0.7469 (6)	−0.1263 (6)	−0.0199 (4)	0.0148 (9)	
O5	0.6365 (6)	−0.1126 (6)	0.2173 (4)	0.0151 (9)	
O6	0.9564 (6)	−0.1194 (5)	0.1274 (4)	0.0129 (9)	
H6O	1.0047	−0.2157	0.1660	0.015*	
O7	0.2360 (6)	0.4303 (6)	0.0624 (4)	0.0145 (9)	
H7O	0.2418	0.3313	0.0485	0.017*	
O8	0.2641 (6)	0.2594 (5)	0.2794 (4)	0.0108 (8)	
O9	0.1203 (6)	0.6039 (6)	0.2530 (4)	0.0141 (9)	
N1	0.6274 (7)	0.2583 (6)	0.2032 (4)	0.0086 (10)	
H1N	0.5590	0.2094	0.2560	0.010*	
C1	0.7944 (8)	0.2553 (8)	0.2523 (6)	0.0116 (12)	
H1A	0.8225	0.3638	0.2177	0.014*	
H1B	0.9018	0.1497	0.2251	0.014*	
C2	0.6768 (8)	0.1763 (8)	0.0791 (5)	0.0089 (11)	
H2A	0.7749	0.2130	0.0257	0.011*	
H2B	0.5677	0.2209	0.0351	0.011*	
C3	0.4836 (8)	0.4433 (8)	0.1986 (6)	0.0111 (12)	
H3A	0.5140	0.5133	0.1209	0.013*	
H3B	0.4828	0.5047	0.2708	0.013*	

### Atomic displacement parameters (Å^2^)

**Table:**

	*U*^11^	*U*^22^	*U*^33^	*U*^12^	*U*^13^	*U*^23^
Tl1	0.00946 (12)	0.01138 (12)	0.01117 (12)	−0.00463 (9)	−0.00049 (9)	−0.00006 (9)
Tl2	0.01643 (14)	0.01862 (14)	0.00967 (13)	−0.00101 (10)	0.00066 (10)	0.00088 (10)
P1	0.0291 (10)	0.0340 (10)	0.0090 (8)	−0.0260 (9)	−0.0018 (7)	0.0005 (7)
P2	0.0088 (7)	0.0092 (7)	0.0072 (7)	−0.0023 (6)	0.0003 (6)	0.0001 (6)
P3	0.0081 (7)	0.0095 (7)	0.0080 (7)	−0.0021 (6)	0.0001 (6)	0.0003 (6)
O1	0.048 (3)	0.031 (2)	0.028 (2)	−0.026 (2)	0.006 (2)	−0.0084 (19)
O2	0.023 (2)	0.051 (3)	0.027 (2)	−0.0233 (19)	−0.0095 (18)	0.0074 (19)
O3	0.011 (2)	0.012 (2)	0.013 (2)	−0.0062 (17)	−0.0001 (18)	0.0018 (17)
O4	0.023 (2)	0.010 (2)	0.011 (2)	−0.0034 (18)	−0.0045 (19)	−0.0010 (17)
O5	0.010 (2)	0.020 (2)	0.011 (2)	−0.0038 (18)	0.0011 (18)	0.0028 (18)
O6	0.010 (2)	0.010 (2)	0.015 (2)	−0.0028 (17)	−0.0031 (18)	0.0090 (17)
O7	0.020 (2)	0.012 (2)	0.011 (2)	−0.0045 (19)	−0.0062 (19)	0.0013 (17)
O8	0.010 (2)	0.009 (2)	0.012 (2)	−0.0033 (17)	0.0013 (17)	−0.0001 (16)
O9	0.011 (2)	0.013 (2)	0.014 (2)	0.0010 (18)	−0.0017 (18)	−0.0027 (18)
N1	0.009 (2)	0.011 (2)	0.007 (2)	−0.005 (2)	0.0031 (19)	−0.0031 (19)
C1	0.009 (3)	0.016 (3)	0.014 (3)	−0.007 (2)	−0.001 (2)	−0.004 (2)
C2	0.006 (3)	0.011 (3)	0.007 (3)	−0.001 (2)	0.002 (2)	−0.001 (2)
C3	0.010 (3)	0.012 (3)	0.009 (3)	−0.003 (2)	0.004 (2)	−0.006 (2)

### Geometric parameters (Å, °)

**Table:**

Tl1—O8	2.555 (4)	P3—C3	1.830 (6)
Tl1—O5	2.569 (4)	O1—Tl2^iii^	2.906 (5)
Tl1—O4^i^	2.778 (4)	O1—H1O	0.8200
Tl1—O3^ii^	3.159 (4)	O2—Tl2^vi^	2.965 (5)
Tl2—O5^ii^	2.870 (4)	O2—H2O	0.8201
Tl2—O8	2.871 (4)	O3—Tl2^ii^	2.926 (4)
Tl2—O1^iii^	2.906 (5)	O3—Tl1^ii^	3.159 (4)
Tl2—O3	2.922 (4)	O4—Tl1^i^	2.778 (4)
Tl2—O3^ii^	2.926 (4)	O5—Tl2^ii^	2.870 (4)
Tl2—O2^iv^	2.965 (5)	O6—H6O	0.8200
Tl2—O9^v^	3.159 (4)	O7—H7O	0.8201
P1—O3	1.501 (4)	O9—Tl2^v^	3.159 (4)
P1—O2	1.522 (5)	N1—C3	1.506 (7)
P1—O1	1.529 (6)	N1—C1	1.510 (7)
P1—C1	1.829 (6)	N1—C2	1.517 (7)
P2—O5	1.502 (5)	N1—H1N	0.8699
P2—O4	1.512 (4)	C1—H1A	0.9900
P2—O6	1.552 (4)	C1—H1B	0.9900
P2—C2	1.823 (6)	C2—H2A	0.9900
P3—O9	1.503 (4)	C2—H2B	0.9900
P3—O8	1.507 (4)	C3—H3A	0.9900
P3—O7	1.576 (4)	C3—H3B	0.9900
			
O8—Tl1—O5	82.65 (14)	O9—P3—O7	109.6 (2)
O8—Tl1—O4^i^	73.72 (13)	O8—P3—O7	112.4 (2)
O5—Tl1—O4^i^	90.64 (13)	O9—P3—C3	105.9 (3)
O8—Tl1—O3^ii^	84.83 (12)	O8—P3—C3	104.2 (3)
O5—Tl1—O3^ii^	80.14 (12)	O7—P3—C3	105.6 (3)
O4^i^—Tl1—O3^ii^	157.62 (11)	P1—O1—Tl2^iii^	140.5 (3)
O8—Tl1—Tl2	42.43 (9)	P1—O1—H1O	131.2
O5—Tl1—Tl2	88.38 (10)	Tl2^iii^—O1—H1O	87.0
O4^i^—Tl1—Tl2	115.70 (8)	P1—O2—Tl2^vi^	142.9 (3)
O3^ii^—Tl1—Tl2	44.31 (7)	P1—O2—H2O	122.8
O5^ii^—Tl2—O8	153.26 (12)	Tl2^vi^—O2—H2O	91.4
O5^ii^—Tl2—O1^iii^	90.93 (14)	P1—O3—Tl2	131.5 (2)
O8—Tl2—O1^iii^	94.48 (13)	P1—O3—Tl2^ii^	121.5 (2)
O5^ii^—Tl2—O3	79.76 (12)	Tl2—O3—Tl2^ii^	106.77 (13)
O8—Tl2—O3	76.90 (12)	P1—O3—Tl1^ii^	95.14 (19)
O1^iii^—Tl2—O3	72.87 (14)	Tl2—O3—Tl1^ii^	91.85 (11)
O5^ii^—Tl2—O3^ii^	76.88 (12)	Tl2^ii^—O3—Tl1^ii^	86.74 (11)
O8—Tl2—O3^ii^	84.01 (11)	P2—O4—Tl1^i^	130.9 (2)
O1^iii^—Tl2—O3^ii^	145.49 (14)	P2—O5—Tl1	130.2 (2)
O3—Tl2—O3^ii^	73.23 (13)	P2—O5—Tl2^ii^	122.6 (2)
O5^ii^—Tl2—O2^iv^	123.69 (14)	Tl1—O5—Tl2^ii^	106.85 (15)
O8—Tl2—O2^iv^	66.41 (13)	P2—O6—H6O	119.1
O1^iii^—Tl2—O2^iv^	137.28 (16)	P3—O7—H7O	111.0
O3—Tl2—O2^iv^	132.04 (14)	P3—O8—Tl1	141.1 (2)
O3^ii^—Tl2—O2^iv^	73.22 (14)	P3—O8—Tl2	118.2 (2)
O5^ii^—Tl2—O9^v^	76.06 (12)	Tl1—O8—Tl2	100.67 (13)
O8—Tl2—O9^v^	129.64 (11)	P3—O9—Tl2^v^	141.9 (3)
O1^iii^—Tl2—O9^v^	92.73 (14)	C3—N1—C1	111.4 (4)
O3—Tl2—O9^v^	151.59 (11)	C3—N1—C2	112.6 (4)
O3^ii^—Tl2—O9^v^	114.72 (11)	C1—N1—C2	112.4 (4)
O2^iv^—Tl2—O9^v^	74.91 (13)	C3—N1—H1N	95.5
O5^ii^—Tl2—Tl1	125.55 (9)	C1—N1—H1N	113.9
O8—Tl2—Tl1	36.90 (8)	C2—N1—H1N	109.9
O1^iii^—Tl2—Tl1	129.09 (10)	N1—C1—P1	110.2 (4)
O3—Tl2—Tl1	79.78 (8)	N1—C1—H1A	109.6
O3^ii^—Tl2—Tl1	48.95 (8)	P1—C1—H1A	109.6
O2^iv^—Tl2—Tl1	52.33 (11)	N1—C1—H1B	109.6
O9^v^—Tl2—Tl1	126.82 (8)	P1—C1—H1B	109.6
O3—P1—O2	115.2 (3)	H1A—C1—H1B	108.1
O3—P1—O1	114.5 (3)	N1—C2—P2	110.9 (4)
O2—P1—O1	108.2 (3)	N1—C2—H2A	109.5
O3—P1—C1	106.0 (3)	P2—C2—H2A	109.5
O2—P1—C1	104.8 (3)	N1—C2—H2B	109.5
O1—P1—C1	107.4 (3)	P2—C2—H2B	109.5
O5—P2—O4	117.4 (3)	H2A—C2—H2B	108.1
O5—P2—O6	111.2 (2)	N1—C3—P3	110.7 (4)
O4—P2—O6	112.0 (3)	N1—C3—H3A	109.5
O5—P2—C2	106.8 (3)	P3—C3—H3A	109.5
O4—P2—C2	104.8 (3)	N1—C3—H3B	109.5
O6—P2—C2	103.2 (3)	P3—C3—H3B	109.5
O9—P3—O8	118.1 (3)	H3A—C3—H3B	108.1
			
O8—Tl1—Tl2—O5^ii^	−151.22 (18)	O5—P2—O4—Tl1^i^	132.5 (3)
O5—Tl1—Tl2—O5^ii^	−70.36 (17)	O6—P2—O4—Tl1^i^	−97.0 (3)
O4^i^—Tl1—Tl2—O5^ii^	−160.29 (15)	C2—P2—O4—Tl1^i^	14.2 (4)
O3^ii^—Tl1—Tl2—O5^ii^	7.15 (15)	O4—P2—O5—Tl1	−51.5 (4)
O5—Tl1—Tl2—O8	80.86 (17)	O6—P2—O5—Tl1	177.6 (3)
O4^i^—Tl1—Tl2—O8	−9.07 (17)	C2—P2—O5—Tl1	65.7 (4)
O3^ii^—Tl1—Tl2—O8	158.37 (18)	O4—P2—O5—Tl2^ii^	136.2 (3)
O8—Tl1—Tl2—O1^iii^	−23.9 (2)	O6—P2—O5—Tl2^ii^	5.3 (3)
O5—Tl1—Tl2—O1^iii^	56.95 (18)	C2—P2—O5—Tl2^ii^	−106.6 (3)
O4^i^—Tl1—Tl2—O1^iii^	−32.99 (19)	O8—Tl1—O5—P2	−77.8 (3)
O3^ii^—Tl1—Tl2—O1^iii^	134.46 (19)	O4^i^—Tl1—O5—P2	−4.3 (3)
O8—Tl1—Tl2—O3	−81.77 (16)	O3^ii^—Tl1—O5—P2	−163.8 (3)
O5—Tl1—Tl2—O3	−0.90 (12)	Tl2—Tl1—O5—P2	−120.0 (3)
O4^i^—Tl1—Tl2—O3	−90.84 (13)	O8—Tl1—O5—Tl2^ii^	95.39 (16)
O3^ii^—Tl1—Tl2—O3	76.60 (15)	O4^i^—Tl1—O5—Tl2^ii^	168.88 (15)
O8—Tl1—Tl2—O3^ii^	−158.37 (18)	O3^ii^—Tl1—O5—Tl2^ii^	9.39 (13)
O5—Tl1—Tl2—O3^ii^	−77.51 (15)	Tl2—Tl1—O5—Tl2^ii^	53.19 (12)
O4^i^—Tl1—Tl2—O3^ii^	−167.44 (15)	O9—P3—O8—Tl1	137.8 (3)
O8—Tl1—Tl2—O2^iv^	100.75 (19)	O7—P3—O8—Tl1	8.7 (5)
O5—Tl1—Tl2—O2^iv^	−178.39 (16)	C3—P3—O8—Tl1	−105.1 (4)
O4^i^—Tl1—Tl2—O2^iv^	91.67 (17)	O9—P3—O8—Tl2	−39.7 (3)
O3^ii^—Tl1—Tl2—O2^iv^	−100.88 (17)	O7—P3—O8—Tl2	−168.8 (2)
O8—Tl1—Tl2—O9^v^	109.29 (17)	C3—P3—O8—Tl2	77.3 (3)
O5—Tl1—Tl2—O9^v^	−169.85 (14)	O5—Tl1—O8—P3	86.5 (4)
O4^i^—Tl1—Tl2—O9^v^	100.22 (15)	O4^i^—Tl1—O8—P3	−6.3 (4)
O3^ii^—Tl1—Tl2—O9^v^	−92.34 (15)	O3^ii^—Tl1—O8—P3	167.2 (4)
O3—P1—O1—Tl2^iii^	173.5 (4)	Tl2—Tl1—O8—P3	−177.8 (5)
O2—P1—O1—Tl2^iii^	43.6 (5)	O5—Tl1—O8—Tl2	−95.68 (14)
C1—P1—O1—Tl2^iii^	−69.0 (5)	O4^i^—Tl1—O8—Tl2	171.49 (16)
O3—P1—O2—Tl2^vi^	−143.4 (4)	O3^ii^—Tl1—O8—Tl2	−14.98 (12)
O1—P1—O2—Tl2^vi^	−13.9 (6)	O5^ii^—Tl2—O8—P3	−121.0 (3)
C1—P1—O2—Tl2^vi^	100.4 (5)	O1^iii^—Tl2—O8—P3	−20.0 (3)
O2—P1—O3—Tl2	144.1 (3)	O3—Tl2—O8—P3	−91.2 (2)
O1—P1—O3—Tl2	17.7 (4)	O3^ii^—Tl2—O8—P3	−165.3 (3)
C1—P1—O3—Tl2	−100.5 (3)	O2^iv^—Tl2—O8—P3	120.4 (3)
O2—P1—O3—Tl2^ii^	−42.3 (4)	O9^v^—Tl2—O8—P3	77.3 (3)
O1—P1—O3—Tl2^ii^	−168.6 (3)	Tl1—Tl2—O8—P3	178.4 (3)
C1—P1—O3—Tl2^ii^	73.1 (3)	O5^ii^—Tl2—O8—Tl1	60.5 (3)
O2—P1—O3—Tl1^ii^	47.0 (3)	O1^iii^—Tl2—O8—Tl1	161.60 (16)
O1—P1—O3—Tl1^ii^	−79.3 (3)	O3—Tl2—O8—Tl1	90.36 (14)
C1—P1—O3—Tl1^ii^	162.5 (2)	O3^ii^—Tl2—O8—Tl1	16.23 (13)
O5^ii^—Tl2—O3—P1	−106.4 (3)	O2^iv^—Tl2—O8—Tl1	−58.05 (16)
O8—Tl2—O3—P1	86.7 (3)	O9^v^—Tl2—O8—Tl1	−101.10 (16)
O1^iii^—Tl2—O3—P1	−12.2 (3)	O8—P3—O9—Tl2^v^	−15.8 (5)
O3^ii^—Tl2—O3—P1	174.3 (4)	O7—P3—O9—Tl2^v^	114.7 (4)
O2^iv^—Tl2—O3—P1	127.0 (3)	C3—P3—O9—Tl2^v^	−131.9 (4)
O9^v^—Tl2—O3—P1	−74.5 (4)	C3—N1—C1—P1	80.8 (5)
Tl1—Tl2—O3—P1	124.3 (3)	C2—N1—C1—P1	−151.7 (4)
O5^ii^—Tl2—O3—Tl2^ii^	79.25 (14)	O3—P1—C1—N1	33.2 (5)
O8—Tl2—O3—Tl2^ii^	−87.61 (13)	O2—P1—C1—N1	155.5 (4)
O1^iii^—Tl2—O3—Tl2^ii^	173.44 (17)	O1—P1—C1—N1	−89.7 (5)
O3^ii^—Tl2—O3—Tl2^ii^	0.0	C3—N1—C2—P2	−152.4 (4)
O2^iv^—Tl2—O3—Tl2^ii^	−47.3 (2)	C1—N1—C2—P2	80.8 (5)
O9^v^—Tl2—O3—Tl2^ii^	111.2 (2)	O5—P2—C2—N1	39.1 (4)
Tl1—Tl2—O3—Tl2^ii^	−50.01 (10)	O4—P2—C2—N1	164.3 (4)
O5^ii^—Tl2—O3—Tl1^ii^	−7.90 (11)	O6—P2—C2—N1	−78.3 (4)
O8—Tl2—O3—Tl1^ii^	−174.76 (12)	C1—N1—C3—P3	−156.6 (4)
O1^iii^—Tl2—O3—Tl1^ii^	86.28 (14)	C2—N1—C3—P3	76.0 (5)
O3^ii^—Tl2—O3—Tl1^ii^	−87.15 (13)	O9—P3—C3—N1	156.2 (4)
O2^iv^—Tl2—O3—Tl1^ii^	−134.49 (15)	O8—P3—C3—N1	31.0 (4)
O9^v^—Tl2—O3—Tl1^ii^	24.0 (3)	O7—P3—C3—N1	−87.6 (4)
Tl1—Tl2—O3—Tl1^ii^	−137.17 (9)		

Symmetry codes: (i) −*x*+1, −*y*, −*z*; (ii) −*x*+1, −*y*, −*z*+1; (iii) −*x*+1, −*y*+1, −*z*+1; (iv) *x*−1, *y*, *z*; (v) −*x*, −*y*+1, −*z*+1; (vi) *x*+1, *y*, *z*.

### Hydrogen-bond geometry (Å, °)

**Table:**

*D*—H···*A*	*D*—H	H···*A*	*D*···*A*	*D*—H···*A*
N1—H1N···O3	0.87	2.39	2.882 (5)	116
N1—H1N···O5	0.87	2.53	2.957 (6)	111
N1—H1N···O8	0.87	2.19	2.837 (7)	131
O1—H1O···O1^iii^	0.82	1.78	2.504 (8)	147
O2—H2O···O2^vii^	0.82	1.68	2.497 (7)	171
O6—H6O···O9^viii^	0.82	1.67	2.484 (6)	171
O7—H7O···O4^i^	0.82	1.70	2.521 (6)	180

Symmetry codes:  (iii) −*x*+1, −*y*+1, −*z*+1; (vii) −*x*+2, −*y*, −*z*+1; (viii) *x*+1, *y*−1, *z*; (i) −*x*+1, −*y*, −*z*.

## References

[bb1] Bruker (2007). *APEX2*, *SAINT *and *XPREP* Bruker AXS Inc., Madison, Wisconsin, USA.

[bb2] Sharma, C. V. K., Clearfield, A., Cabeza, A., Aranda, M. A. G. & Bruque, S. (2001). *J. Am. Chem. Soc* **123**, 2885–2886. 10.1021/ja0032430 11456977

[bb3] Sheldrick, G. M. (2008). *Acta Cryst.* A**64**, 112–122. 10.1107/S0108767307043930 18156677

